# Advances in the Prevention and Management of Celiac Disease

**DOI:** 10.3390/nu17132240

**Published:** 2025-07-07

**Authors:** Teresa Nestares, Rafael Martín-Masot

**Affiliations:** 1 Department of Physiology, Faculty of Pharmacy, University of Granada, 18071 Granada, Spain; 2 Pediatric Gastroenterology and Nutrition Unit, Hospital Regional Universitario de Malaga, 29010 Málaga, Spain; rafammgr@gmail.com; 3 Department of Pharmacology and Pediatrics, School of Medicine, University of Málaga, 29010 Málaga, Spain

Celiac disease (CD) exemplifies the complexity inherent in multifactorial, immune-mediated disorders, with layers of pathogenesis and clinical expression that continue to challenge both researchers and clinicians. Rather than a straightforward food intolerance, CD is now recognized as a systemic autoimmune disease with a heterogeneous clinical spectrum, capable of affecting any organ system and manifesting in both gastrointestinal and extraintestinal symptoms [1,2]. Over recent decades, the global prevalence of CD has steadily increased, accompanied by growing awareness of its diagnostic challenges, a broadening range of comorbidities, and the persistent need for improved strategies in both prevention and management [3,4].

Central to current research is the recognition that while gluten exposure is essential for the development of CD, it acts within a complex network of genetic and environmental factors [5]. Genetic predisposition—particularly HLA-DQ2 and DQ8—remains a fundamental requirement, yet disease susceptibility and clinical course are shaped by additional elements such as the timing and quantity of gluten introduction, the gut microbiota composition, dietary patterns, infectious exposures, impairment of the intestinal barrier, and maladaptive immune responses [6,7,8,9,10]. This intricate interplay highlights the urgency of refining our understanding of disease mechanisms, enhancing diagnostic accuracy, and developing innovative management strategies that extend beyond the gluten-free diet (GFD).

This Special Issue of *Nutrients* brings together eight original research articles and reviews that reflect the current State of the Art in CD, with a particular focus on environmental determinants and the ongoing search for novel preventive and therapeutic approaches. The collection includes comprehensive analyses of public health interventions and policy, such as a multicentric evaluation of how policies for celiac disease can transform patient lives across diverse settings (Contribution 1), and a nationwide assessment of eating attitudes and disordered eating risk in adults with CD in Brazil (Contribution 2). The nutritional landscape is further explored in pediatric and adolescent populations, with a comparative study of breakfast quality and the role of gluten-free products among Spanish children with and without CD (Contribution 3), as well as an investigation into the importance of early nutritional evaluation following the initiation of a GFD in children (Contribution 4).

Diagnostic innovation and monitoring are at the forefront in this issue, exemplified by the development and validation of a novel automated immunoassay for urinary immunogenic gluten peptides as a marker of dietary adherence (Contribution 5). The spectrum of clinical presentation is addressed in a large multicenter case–control study exploring the clinical, serological, and genetic differences between symptomatic and asymptomatic patients, raising important questions about current risk-based screening strategies (Contribution 6). The extraintestinal manifestations of CD are also considered, with a narrative review on the impact of gluten and CD on male and female reproductive health (Contribution 7), expanding our perspective on the systemic effects of the disease.

Finally, the Special Issue underscores the transformative potential of omics sciences—particularly metabolomics—in CD research and clinical care. A State-of-the-Art review explores how metabolomic profiling is shedding light on early diagnosis, disease monitoring, and the molecular impact of the GFD, pointing toward a future of precision medicine and targeted interventions (Contribution 8).

Together, these contributions highlight the necessity of a multidisciplinary, evidence-based, and technologically advanced approach to CD. The future of research and clinical care in CD will depend on collaboration among clinicians, nutritionists, molecular scientists, and data specialists, all working to translate molecular insights into meaningful patient outcomes.

We are deeply grateful to all authors, reviewers, and the editorial staff at *Nutrients* for their dedication to this Special Issue. It is our hope that the research presented here will foster further innovation, inspire new collaborations, and ultimately advance the care and quality of life of all individuals living with CD.
